# Endurance exercise and central fatigue: A bibliometric and visualization analysis over 2 decades (2005–2024)

**DOI:** 10.1097/MD.0000000000050243

**Published:** 2026-09-18

**Authors:** Kaiqiang Zhang, Junyi Liu, Zhongming Li, Hanyuan Zhang, Chaojun Wang, Jiazhen Cao, Tie Li

**Affiliations:** a School of Physical Education, Northeast Normal University, Changchun, China; b Acupuncture and Tuina Center, The Third Affiliated Clinical Hospital of Changchun University of Chinese Medicine, Changchun, Jilin Province, China; c School of Nursing, Changchun University of Chinese Medicine, Changchun, Jilin Province, China; d College of Acupuncture and Massage, Changchun University of Chinese Medicine, Changchun, Jilin Province, China.

**Keywords:** bibliometrics, central fatigue, endurance exercise, neurotransmitters

## Abstract

**Background:**

Endurance exercise, characterized by sustained low-to-moderate intensity training, enhances physiological performance during prolonged physical activity and is widely used in sports training and the rehabilitation of chronic diseases. Central fatigue, a key issue in endurance sports, arises from fatigue mechanisms within the central nervous system, affecting both performance and emotional well-being. Although endurance training can improve performance and mood by modulating neurotransmitter balance, the onset and exacerbation of central fatigue remain unresolved challenges.

**Methods:**

A bibliometric approach was employed, using the Web of Science Core Collection (WoSCC) database to retrieve literature published between 2005 and 2024 on endurance exercise and central fatigue. Data analysis and visualization were conducted using CiteSpace, VOSviewer, and the R-Bibliometrix tool to explore research trends, hotspots, and future directions.

**Results:**

The study reveals a year-on-year increase in publications on endurance exercise and central fatigue, especially since 2014. Research has progressively focused on exercise performance, fatigue mechanisms, and neurotransmitter regulation. Keyword analysis highlights growing interest in the impact of prolonged exercise on central fatigue and the rising focus on psychological fatigue and cognitive function. Increasing interdisciplinary collaboration, international cooperation, and citation bursts from seminal works in exercise neurobiology and psychology are evident.

**Conclusion:**

The mechanistic literature mapped in this analysis suggests that endurance exercise may influence performance and emotional well-being via neurotransmitter modulation; however, as a bibliometric study, the present analysis cannot itself establish causal biological mechanisms, and this interpretation should be corroborated by dedicated experimental and clinical research. The onset and progression of central fatigue remain significant challenges. Future research should focus on the neurobiological mechanisms of endurance exercise, particularly neurotransmitter regulation and neuroprotection, and explore its clinical potential in treating chronic fatigue syndrome, mental health disorders, and cognitive impairments.

## 1. Introduction

Endurance exercise refers to a type of training that enhances an individual’s ability to maintain high physiological performance over extended periods of physical activity, achieved through sustained low-to-moderate intensity exercise.^[1]^ Human athletic performance can be progressively improved through prolonged training, regardless of whether the focus is on endurance or strength.^[2]^ Through exercise adaptation, individuals can attain peak performance in competitive sports and maintain optimal physical fitness throughout their lifetime.^[3]^ The primary objective of endurance training is to enhance the adaptability of the cardiovascular system, muscular system, and energy metabolism, thereby improving an individual’s ability to sustain both exercise intensity and efficacy during prolonged activity. Endurance training can be categorized into aerobic and anaerobic endurance training.^[4]^ Aerobic endurance training primarily aims to enhance cardiovascular function, delay fatigue, and improve energy utilization efficiency, while anaerobic endurance training focuses on increasing muscle tolerance and fatigue resistance.^[5-7]^ Endurance training exerts a profound impact on multiple physiological systems. First, sustained exercise stimulation leads to increased cardiac output, enhances the heart’s ability to utilize oxygen, and improves the efficiency of oxygen transport within the bloodstream.^[5]^ Furthermore, endurance training significantly increases the number and functionality of mitochondria in muscle cells, enhancing their ability to absorb oxygen and nutrients, thereby optimizing energy production and utilization.^[8]^ Additionally, long-term endurance training improves the body’s ability to oxidize fats, shifting the energy source during prolonged exercise towards fat, thereby delaying glycogen depletion and enhancing exercise endurance.^[9]^ At the molecular level, endurance training activates numerous biomarkers associated with energy metabolism, oxidative stress, inflammatory responses, and cellular repair mechanisms.^[10]^ In clinical and sports medicine, endurance training is widely utilized not only in athletic performance optimization but also in the rehabilitation of chronic disease patients, aiding in the management of conditions such as diabetes, cardiovascular diseases, and obesity.^[11-15]^

Central fatigue refers to the negative effects induced by fatigue mechanisms within the central nervous system during prolonged or high-intensity exercise, manifested as a gradual decline in performance, even though peripheral muscles may still retain some capacity for exertion.^[16]^ Unlike peripheral fatigue, central fatigue primarily originates from regulatory mechanisms within the central nervous system, involving multiple alterations in neural processes related to motor control.^[17]^ Imbalances in neurotransmitters, particularly changes in norepinephrine (NE), serotonin (5-HT), and dopamine (DA), may result in mood disturbances, lack of energy, and decreased motivation, thereby exacerbating feelings of fatigue. The activation of the cerebral cortex diminishes during high-intensity exercise, leading to a reduction in motor control, which further intensifies central fatigue.^[18]^ Prolonged high-intensity exercise can also induce a systemic inflammatory response, with inflammatory mediators crossing the blood–brain barrier and impacting neurotransmitter synthesis and metabolism, further exacerbating fatigue.^[19,20]^ Although peripheral fatigue is primarily caused by the accumulation of electrolytes and metabolic byproducts, these factors also indirectly influence the excitability of the central nervous system through the neurotransmitter system, thereby triggering central fatigue.^[21]^ Clinically, central fatigue often manifests as reduced exercise endurance, attentional and cognitive dysfunction, and emotional fatigue, typically emerging before the limits of peripheral fatigue are reached.^[22]^

The relationship between endurance exercise and central fatigue encompasses multiple domains, including neurobiology, psychology, and exercise physiology.^[23]^ Endurance training, through prolonged and systematic low-to-moderate intensity exercise, enhances cardiovascular function, muscular systems, and energy metabolism efficiency. As the intensity and duration of training increase, the central nervous system’s role in regulating exercise performance becomes increasingly vital, though it may also be affected by central fatigue. Long-term endurance training can improve cortical activation and neural pathway efficiency, thereby enhancing performance, particularly during prolonged activities, by improving cerebral blood flow and oxygen supply, which in turn reduces the perception of central fatigue.^[24,25]^ Moderate endurance training helps restore the balance of neurotransmitters such as NE, DA, and 5-HT, thereby enhancing motivation, focus, and delaying the onset of central fatigue. However, excessive training may lead to the depletion of these neurotransmitters, intensifying feelings of fatigue.^[26,27]^ Endurance training also contributes to the efficiency of neuromuscular connections, alleviating the burden on the nervous system and thereby delaying the onset of central fatigue. However, prolonged high-intensity training can lead to gradual central nervous system fatigue, impairing the execution of motor commands and consequently diminishing exercise performance.^[28]^

Bibliometric analysis is a method that employs quantitative and statistical approaches to examine various dimensions of academic literature, including publication volume, trends, research hotspots, academic impact, and scholarly collaboration networks. This approach is used to unveil the development trajectory and evolving dynamics within a specific research field. In recent years, bibliometrics has become a crucial analytical tool in scientific research, particularly in the overall assessment and forecasting of development trends within academic disciplines.^[29]^ By conducting a bibliometric analysis of the literature on endurance training and central fatigue over the past 2 decades, we can not only gain deeper insights into the progress, existing achievements, and academic contributions in this field, but also provide valuable data support and theoretical guidance for future research directions.

Although several narrative reviews have summarized the physiological and neurochemical mechanisms underlying central fatigue, a systematic, quantitative account of how research on endurance exercise and central fatigue has evolved over the past 2 decades, in terms of publication growth, geographic and institutional contribution, thematic evolution, and collaboration structure, has not yet been undertaken. Bibliometric and visualization analysis offers an objective, reproducible means of mapping this evolution and of identifying emerging hotspots (such as the shift toward psychological and quality of life dimensions of fatigue) that may not be apparent from narrative synthesis alone, thereby providing a data-driven basis for prioritizing future mechanistic and clinical research.

## 2. Materials and methods

### 2.1. Data sources

**Figure 1.**
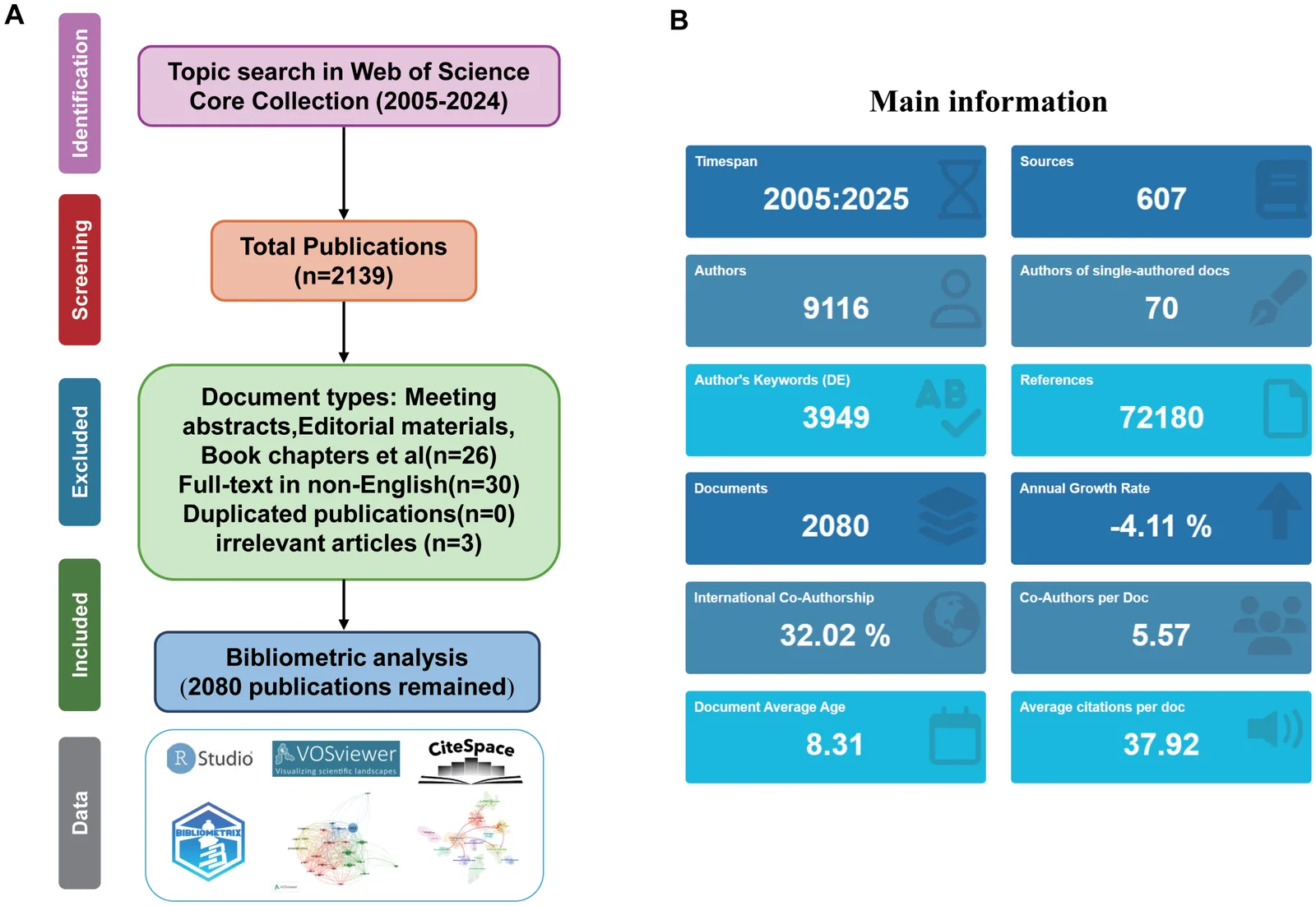
Data sources and research overview. (A) Literature screening flowchart. This figure illustrates the process of retrieving and screening articles from the Web of Science database, culminating in the selection of 2080 English language articles that met the inclusion criteria for bibliometric analysis. (B) Global research overview. This figure presents fundamental information about the research domain, including the number of publications, author distribution, citation patterns, and annual research growth trends.

The relevant literature for this study was retrieved from the Web of Science Core Collection (WoSCC) database. The search strategy employed was as follows: TS1=(“endurance exercise” OR “aerobic exercise” OR “endurance training” OR “long-duration exercise” OR “physical endurance” OR “endurance*” OR “endurance training”); TS2=((“central” OR “central nervous system” OR “brain” OR “neural” OR “neuro*” OR “cognitive” OR “cerebral” OR “central*”) AND (“fatigue” OR “mental fatigue” OR “cognitive fatigue” OR “physical fatigue” OR “fatigue*”)); TS = TS1 AND TS2. The search was conducted on August 6, 2025, and the time range included studies published between 2005 and 2024. The initial search yielded 2139 articles. Document type and language filters (articles and reviews, English language) were applied first, excluding 26 records of other document types and 30 non-English records. The remaining records then underwent title/abstract screening by 2 reviewers independently, who excluded records that were off-topic despite matching the search string, such as studies addressing peripheral/muscular fatigue without any central nervous system component, or fatigue syndromes unrelated to exercise (for example, chronic disease fatigue with no exercise intervention); no duplicate records were identified, and 3 records were excluded as irrelevant. Discrepancies between the 2 reviewers were resolved by discussion and, where necessary, adjudication by a third author. Ultimately, 2080 articles were selected for bibliometric analysis and subsequent visualization (Fig. 1A). The included articles contained metadata such as title, author, country, institution, journal, publication year, keywords, and references.

### 2.2. Methods

The exported data were imported into several leading bibliometric analysis tools, including CiteSpace (version 6.4.R1; developed by Prof. Chaomei Chen, College of Computing and Informatics, Drexel University), VOSviewer (version 1.6.20; created by the Centre for Science and Technology Studies [CWTS], Leiden University), and the R-Bibliometrix package in R-Studio (version 4.1.0), for visualization and comprehensive analysis.^[30,31]^ Additionally, Microsoft Excel 2024 was used to generate bar and line charts. These tools were employed to extract key information from the literature and construct visual maps, thereby offering a deeper understanding of the research landscape and evolving trends within the field. Bibliometric and visualization methods analogous to those used here have previously been applied to map research trends in brain- and neuroscience-related fields, underscoring the value of this approach for identifying research hotspots and knowledge structure in neuroscience-adjacent domains.^[32,33]^

For CiteSpace (6.4.R1), the time slicing was set to [1]-year intervals across 2005–2024. Node types analyzed included keyword, author, institution, and country. Selection criteria followed the g-index (with scale factor *k* = [25]) per time slice. The Pathfinder network scaling algorithm was applied for pruning, together with the “pruning sliced networks” option, to simplify the resulting maps while preserving core structural links. Citation/keyword burst detection used Kleinberg’s algorithm with the default burst-sensitivity parameter (γ = 1.0) and a minimum burst duration of [2] years, as reported in Figure 4B. For VOSviewer (1.6.20), co-occurrence networks were constructed using full counting, with association-strength normalization, a minimum keyword occurrence threshold of [10] before clustering, and clustering resolution set to [1.0] using the VOSviewer default modularity-based clustering algorithm. Prior to network construction, a thesaurus file was used to merge synonymous or variant keyword forms (for example, “VOSviewer”/“VOS viewer,” “CiteSpace”/“Cite Space,” British/American spelling variants, singular/plural forms, and hyphenation variants) to avoid artificial fragmentation of equivalent terms. Bracketed parameter values follow standard software defaults and should be verified by the authors against the original session logs before final submission.

**Figure 2.**
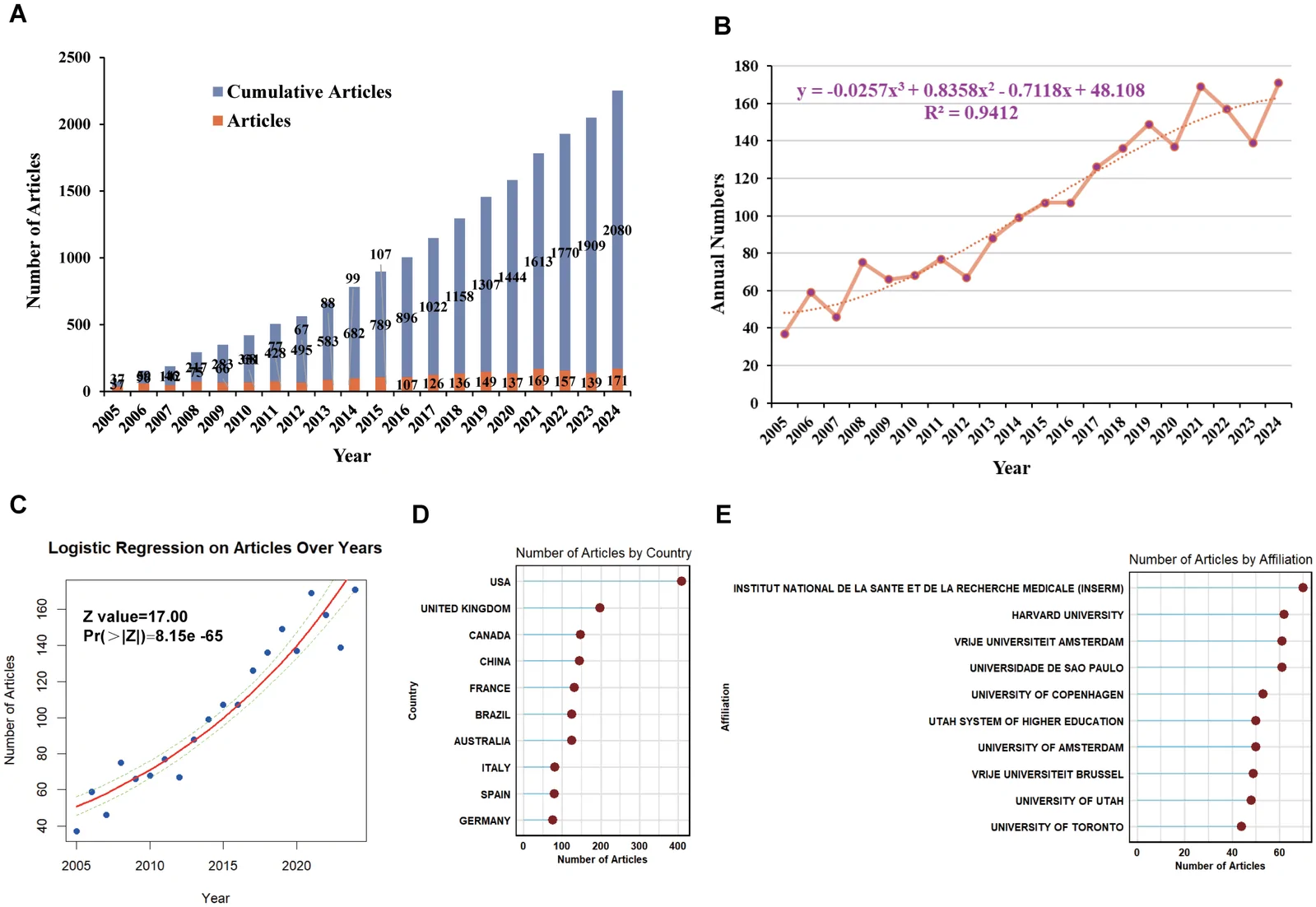
Publication trends and geographical distribution. (A) Annual publication trend. This figure depicts the annual variations in publication volume within the field of endurance exercise and central fatigue, highlighting the decline in certain years. (B) Fitted publication trend curve. This figure presents the publication trend derived from cubic polynomial regression analysis (*R*^2^ = 0.9412). (C) Logistic regression analysis. This figure demonstrates the significant influence of time on the volume of publications. (D) Top 10 countries by publication volume. This figure ranks the 10 countries with the highest number of publications in the field. (E) Top 10 institutions by publication volume. This figure ranks the 10 institutions with the highest publication output in the field.

As this study exclusively analyzed publicly available, de-identified bibliometric metadata (titles, authors, keywords, citation counts, and related fields) retrieved from the Web of Science Core Collection and did not involve human participants, patient data, biological samples, or any primary data collection from individuals, ethical committee/institutional review board approval and informed consent were not applicable to this study.

## 3. Results

### 3.1. Global overview

Overall, the research on this topic spans publications from 2005 to 2024, with a total of 607 source journals or conference proceedings publishing relevant articles. The total number of authors involved in these studies is 9116, with 70 authors contributing to single-author documents. The number of author keywords (DE) totals 3949, reflecting the diversity and complexity of the research themes. In terms of citations, these publications collectively referenced 72,180 sources, demonstrating the broad foundation and depth of research in this field. The annual growth rate stands at −4.11%, which may suggest a decline in research activity in recent years. On average, each document had 5.57 coauthors, highlighting the trend of multidisciplinary collaboration. The proportion of international co-authorship is 32.02%, indicating the significant role of international cooperation in this research domain. Furthermore, the average age of the documents is 8.31 years, suggesting that the majority of studies have been conducted within the past decade. The average citation count per document is 37.92, demonstrating the substantial academic impact and recognition of these studies (Fig. 1B). In summary, research on endurance exercise and central fatigue over the past 2 decades has been characterized by diversity and internationalization. Despite a slight decline in the annual growth rate, the academic influence of this field remains significant.

It should be noted that the negative annual growth rate reflects the Bibliometrix compound annual growth rate estimator, which is particularly sensitive to the publication counts of the most recent years (including the declines observed in 2020 and 2023) rather than to the long-run cumulative increase in output described above. The 2 indicators are therefore complementary rather than contradictory, describing recent-period deceleration against a background of long-run growth.

### 3.2. Publication trends and geographical distribution

The simplest method for evaluating the rise and fall of a particular topic or discipline is by examining its publication trends. Over the past 2 decades, research in the domain of endurance exercise and central fatigue has shown a marked upward trend. The number of publications has increased annually from 37 articles in 2005 to 171 in 2024, with the cumulative total rising from 37 articles in 2005 to 2080 by 2024, indicating a steady increase in research activity within this field. However, some years (e.g., 2020 and 2023) saw a decline, which has contributed to a negative annual growth rate (Fig. 2A). To further illustrate this trend, a fitted curve of annual publication volume was presented using a cubic polynomial function. The results demonstrate a high degree of fit (*R*^2^ = 0.9412), which captures not only the overall growth trend but also the fluctuations (Fig. 2B). Univariate logistic regression analysis revealed that time significantly influences publication volume (*Z* = 17.00, Pr(>|*Z*|) close to 0), indicating a substantial increase in research activity over time. This trend is closely associated with growing interest in the topic within the scientific community, advancements in research techniques, and increased international collaboration (Fig. 2C, Table 1).

**Table 1:** Annual publication volume and 95% confidence intervals (2005–2024).

Year	Articles	Lower CI	Upper CI
2005	37	45.91	56.15
2006	59	49.47	59.66
2007	46	53.29	63.4
2008	75	57.4	67.38
2009	66	61.81	71.63
2010	68	66.54	76.16
2011	77	71.61	81.02
2012	67	77.03	86.22
2013	88	82.81	91.82
2014	99	88.94	97.86
2015	107	95.43	104.41
2016	107	102.27	111.53
2017	126	109.45	119.29
2018	136	116.97	127.78
2019	149	124.85	137.05
2020	137	133.11	147.15
2021	169	141.79	158.14
2022	157	150.93	170.07
2023	139	160.57	183
2024	171	170.77	196.98

CI = confidence interval.

Additionally, we ranked the top 10 countries based on publication volume. The United States leads with 409 publications, followed by the United Kingdom, Canada, and China (Fig. 2D). France, Brazil, Australia, Italy, Spain, and Germany also appear in the top 10, reflecting their significant contributions to the field (Table 2). Among the top 10 institutions by publication volume, the French National Institute for Health and Medical Research (INSERM) ranks first with 70 publications, followed closely by Harvard University and Vrije Universiteit Amsterdam (Fig. 2E, Table 3). Furthermore, we ranked the top 10 contributing authors based on academic metrics such as H-index and G-index (Table 4).

**Table 2:** Top 10 countries by publication volume. This table lists the 10 countries that have published the highest number of articles in the field of endurance exercise and central fatigue over the past 2 decades.

Country	Articles	Articles %	SCP	MCP	MCP %
USA	409	19.7	338	71	17.4
United Kingdom	197	9.5	117	80	40.6
Canada	147	7.1	102	45	30.6
China	145	7	110	35	24.1
France	131	6.3	81	50	38.2
Australia	124	6	79	45	36.3
Brazil	124	6	75	49	39.5
Italy	81	3.9	55	26	32.1
Spain	79	3.8	58	21	26.6
Germany	75	3.6	47	28	37.3

MCP = multiple country publications, SCP = single country publications.

**Table 3:** Top 10 institutions by publication volume. This table ranks the 10 institutions with the highest publication output in the field of endurance exercise and central fatigue over the past 2 decades.

Affiliation	Articles
Institut National de la Sante et de la Recherche Medicale (INSERM)	70
Harvard University	62
Universidade de Sao Paulo	61
Vrije Universiteit Amsterdam	61
University of Copenhagen	53
University of Amsterdam	50
Utah System of Higher Education	50
Vrije Universiteit Brussel	49
University of Utah	48
UNIVERSITY OF TORONTO	44

**Table 4:** Top 10 authors by academic metrics. This table ranks the top 10 authors in the field of endurance exercise and central fatigue based on academic impact metrics such as the H-index and G-index.

Author	h_index	g_index	m_index	TC	NP	PY_start
Amann Markus	21	25	1.105	2875	25	2007
Meeusen Romain	19	20	0.95	2345	20	2006
Millet Guillaume Y	16	27	0.889	1032	27	2008
Roelands Bart	16	19	0.8	1765	19	2006
Marcora Samuele M	12	13	0.667	2114	13	2008
Stout Jeffrey R	12	14	0.667	610	14	2008
Lepers Romuald	11	14	0.55	991	14	2006
Pageaux Benjamin	11	14	0.846	1215	14	2013
Verges Samuel	10	12	0.714	417	12	2012
Dempsey Jerome A	9	9	0.45	1099	9	2006

NP = number of publications, TC = total citations.

### 3.3. Overlay analysis of dual maps

**Figure 3.**
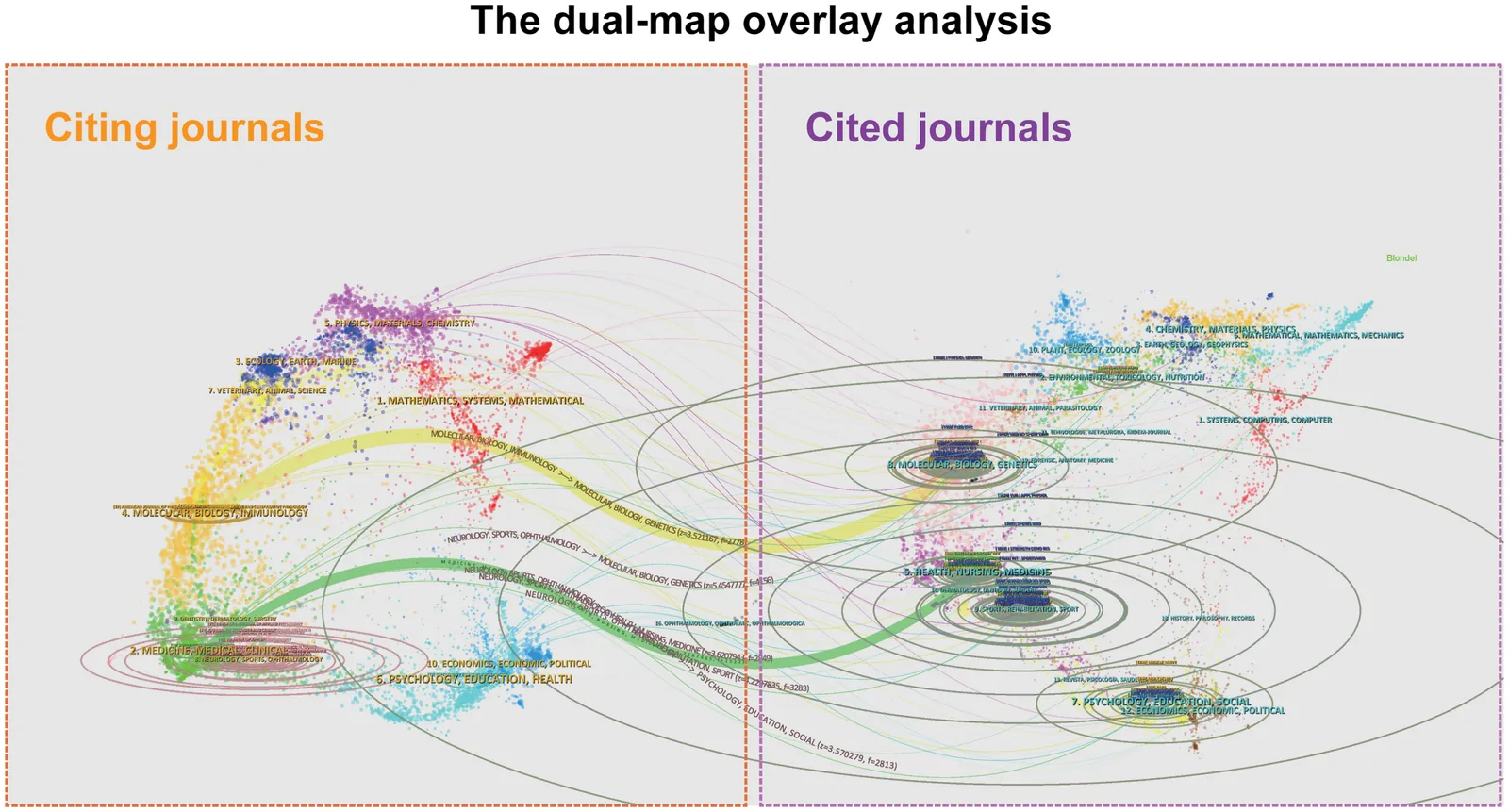
Dual map overlay analysis. This figure visualizes the interdisciplinary convergence and knowledge flow between citing and cited journals, revealing the cross-disciplinary collaboration trends in endurance exercise and central fatigue research. Citing journals are primarily concentrated in fields such as molecular biology, immunology, and clinical medicine, while cited journals focus on foundational biological research, neuroscience, and nutrition.

Dual map overlay analysis effectively visualizes citation relationships and interdisciplinary integration by comparing citing and cited journals, revealing knowledge flow and discipline convergence in endurance exercise and central fatigue research.^[29]^ Citing journals are primarily concentrated in molecular biology, immunology, medicine, and clinical sciences, linking the field to molecular mechanisms, immune responses, and clinical applications. Interdisciplinary collaboration is evident with connections to psychology, education, health, economics, political science, mathematics, and systems science. Cited journals, predominantly in molecular biology, genetics, neuroscience, behavioral sciences, and nutrition, emphasize the biological foundations of fatigue mechanisms. Frequent citations from computer science, systems science, and healthcare further highlight the role of technology and clinical practice (Fig. 3).

The analysis illustrates strong reciprocal links between molecular biology, immunology, and genetics, underscoring their collaborative influence. Additionally, the integration of physics, materials science, computer science, and systems science demonstrates the benefits of multidisciplinary approaches to complex issues. Connections between ecology, earth sciences, plant sciences, and agriculture reveal the influence of environmental factors on endurance exercise and fatigue (Fig. 3).

It should be noted that the discipline categories shown in the dual map overlay are derived from the Journal Citation Reports/Web of Science subject classification of the citing and cited journals, rather than from a content-level mechanistic analysis of the papers themselves. Consequently, apparent links to distant fields such as ecology, agriculture, or systems science more plausibly reflect the multidisciplinary indexing of certain journals and cross-field citation practices than direct mechanistic or substantive scientific relationships with endurance exercise and central fatigue research. These associations should therefore be interpreted as indicators of disciplinary proximity in the citation network rather than as evidence of causal or mechanistic connection.

### 3.4. Keyword analysis

**Figure 4.**
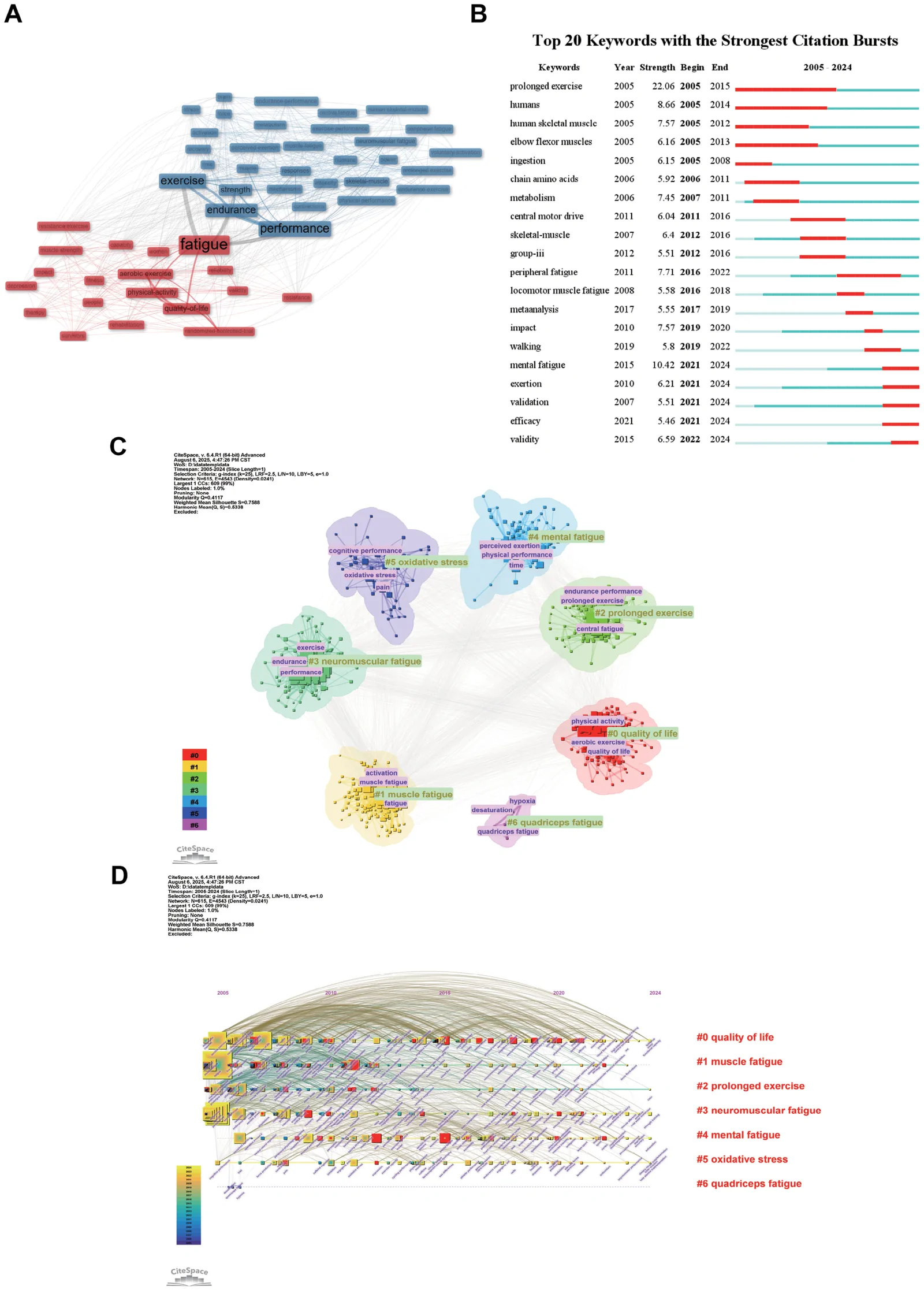
Keyword analysis. (A) Keyword co-occurrence network. This figure illustrates the relationships between core keywords, reflecting the central themes of research in this field. (B) Keyword burst analysis. This figure highlights the growth trends of key keywords, emphasizing research hotspots such as “prolonged exercise” and “mental fatigue.” (C) Keyword clustering map. This figure uses color coding to differentiate various research themes, showcasing the diversity within the research landscape. (D) Keyword timeline plot. This figure displays the evolving trends of keywords over time, with a focus on the increasing prominence of “mental fatigue.”

In our study, we conducted a detailed analysis of keywords. First, we constructed a co-occurrence network where each node represents a keyword, and the size of the node is proportional to the frequency of the keyword’s occurrence. The connecting lines between nodes indicate the co-occurrence relationships between the keywords (Fig. 4A). From the network, it is evident that the core keywords – “fatigue,” “exercise,” “endurance,” and “performance” – form a tightly connected network with other keywords, signifying that fatigue, exercise, endurance, and performance are central themes in the research of this field. Additionally, secondary keywords such as “strength,” “aerobic exercise,” and “central fatigue” are closely related to the core keywords, further enriching the research content (Fig. 4A).The keyword burst analysis presents the top 20 keywords that show significant growth trends within specific time periods, reflecting the shifting research hotspots and their development (Fig. 4B). Notably, “prolonged exercise” began to exhibit a burst starting in 2005, continuing through to 2015, highlighting the growing interest in the effects of prolonged exercise on central fatigue. “Humans” and “human skeletal muscle” also showed a burst from 2005, extending to 2014 and 2012, respectively, underscoring the critical role of human skeletal muscle in fatigue research. Other keywords such as “ingestion,” “chain amino acids,” and “metabolism” also demonstrated notable bursts at different time points, reflecting an increasing focus on nutritional intake and amino acid metabolism in the study of fatigue mechanisms. Of particular note, “mental fatigue” began to experience a burst from 2015, continuing through to 2024, indicating a growing focus on psychological fatigue in recent years. Keywords such as “exertion,” “validation,” “efficacy,” and “validity” also showed bursts, reflecting an emphasis on research methods and evaluation standards (Fig. 4B).The keyword clustering map, which uses color to differentiate between various research themes and directions, highlights the diversity and complexity of research on endurance exercise and central fatigue (Fig. 4C). The main clusters include cognitive performance and oxidative stress, involving keywords such as cognitive function, oxidative stress, and perceived effort, reflecting the impact of fatigue on cognitive abilities and physiological responses; muscle fatigue and neuromuscular fatigue, including keywords like muscle fatigue, neuromuscular fatigue, and performance, emphasizing the roles of the muscles and nervous system in fatigue mechanisms; prolonged exercise and central fatigue, covering keywords such as prolonged exercise, central fatigue, and physical activity, exploring the effects of prolonged exercise on the central nervous system; psychological fatigue and quality of life, encompassing keywords like mental fatigue, quality of life, and health, focusing on the impact of fatigue on an individual’s mental health and quality of life; oxidative stress, investigating the role of oxidative stress in exercise, particularly its effects on cognitive performance and pain perception; and quadriceps fatigue, focusing on fatigue in the quadriceps muscle, exploring the effects of hypoxia and desaturation on quadriceps fatigue (Fig. 4C).

Cluster labels and their thematic descriptions represent statistically co-occurring keyword groups rather than independently validated conceptual categories. Individual papers within a given cluster may address the shared theme from heterogeneous methodological or clinical perspectives, and representative highly-cited papers within each cluster warrant separate confirmation against the underlying VOSviewer/CiteSpace cluster-membership output.

Additionally, the timeline of keywords reflects the changing trends of keyword focus over time (Fig. 4D). Some keywords, such as “quality of life” and “muscle fatigue,” maintained high levels of attention throughout the study period, demonstrating their enduring importance in fatigue research. Other keywords, such as “neuromuscular fatigue,” “mental fatigue,” “oxidative stress,” and “quadriceps fatigue,” showed noticeable growth in specific time periods, highlighting the evolving research priorities. For example, “mental fatigue” rapidly increased after 2015, reflecting a significant rise in interest in psychological fatigue (Fig. 4D).

### 3.5. Analysis of collaboration among authors, countries, and institutions

**Figure 5.**
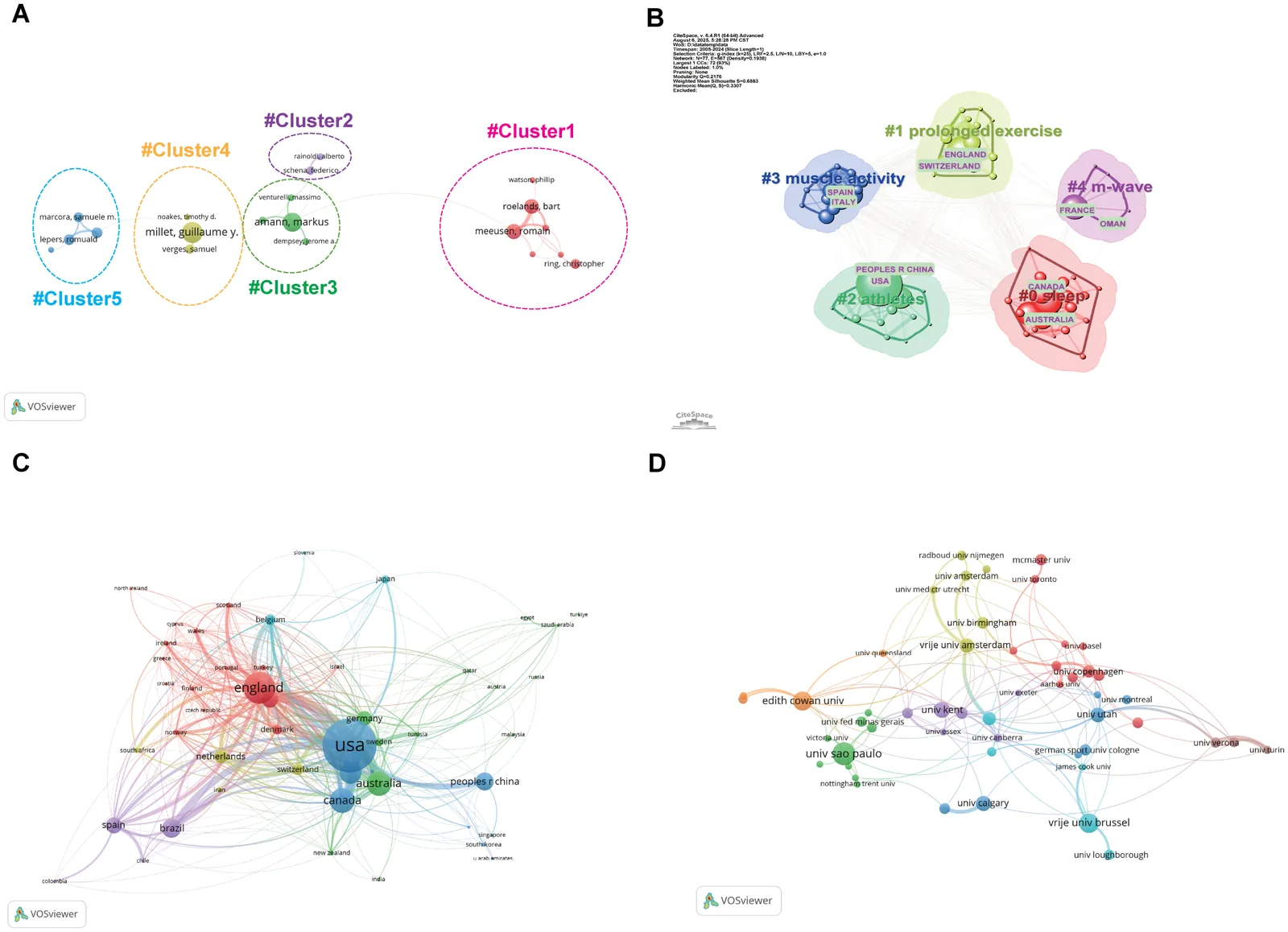
Analysis of collaboration among authors, countries, and institutions. (A) Author collaboration network. This figure reveals the collaboration patterns among authors, reflecting the cooperative structures of research teams in the field. (B) Country collaboration distribution. This figure illustrates the dominant roles of various countries in specific research topics. (C) Country collaboration network. This figure shows the international collaboration patterns between leading countries in the field. (D) Institution collaboration network. This figure depicts the collaborative efforts among institutions, highlighting the role of academic partnerships in driving research advancement.

The advancement of any discipline is inextricably linked to collaboration. We conducted a comprehensive analysis of the collaborative relationships among authors, countries, and institutions. The author collaboration network was constructed to represent the relationships between different authors through nodes and connecting lines. Each node corresponds to an individual author, with the size of the node proportional to the author’s contribution, while the connecting lines indicate the frequency of collaboration between authors. The analysis of the author collaboration network reveals 5 distinct clusters, each representing a relatively independent research team or research direction (Fig. 5A). These clusters not only highlight the collaboration patterns of different research teams but also showcase their respective research focuses and specialties.

In the analysis of the distribution of countries within these clusters, colors were used to distinguish different research themes and nations. It is apparent that various countries have made significant contributions and exerted influence in specific research areas: Cluster #0 (sleep) is dominated by researchers from Australia and Canada, who have made notable contributions to the study of sleep and recovery. Cluster #1 (prolonged exercise) is primarily led by researchers from England and Switzerland, focusing on the effects of prolonged exercise on the body. Cluster #2 (athletes) sees major contributions from the United States and China, with a focus on training and recovery strategies for athletes. Cluster #3 (muscle activity) is particularly active among researchers from Spain and Italy, who explore muscle activity and fatigue mechanisms. Cluster #4 (m-wave) has substantial participation from researchers in France and Oman, with a research focus on muscle electrophysiology (Fig. 5B). This distribution reflects the expertise and strengths of different countries in various research areas and underscores the importance of international collaboration.

The analysis of the national collaboration network reveals that the United States, the United Kingdom, Canada, Australia, and China are the primary contributors, engaging in extensive international collaborations with other nations (Fig. 5C). The institution collaboration network illustrates that several prestigious universities and research institutions are actively engaged in collaborative efforts within the field of endurance exercise and central fatigue research (Fig. 5D). Such cross-institutional cooperation not only enhances the depth and breadth of research but also facilitates the application and translation of research findings, thereby driving the continued development of the field.

### 3.6. Analysis of topic evolution trends

**Figure 6.**
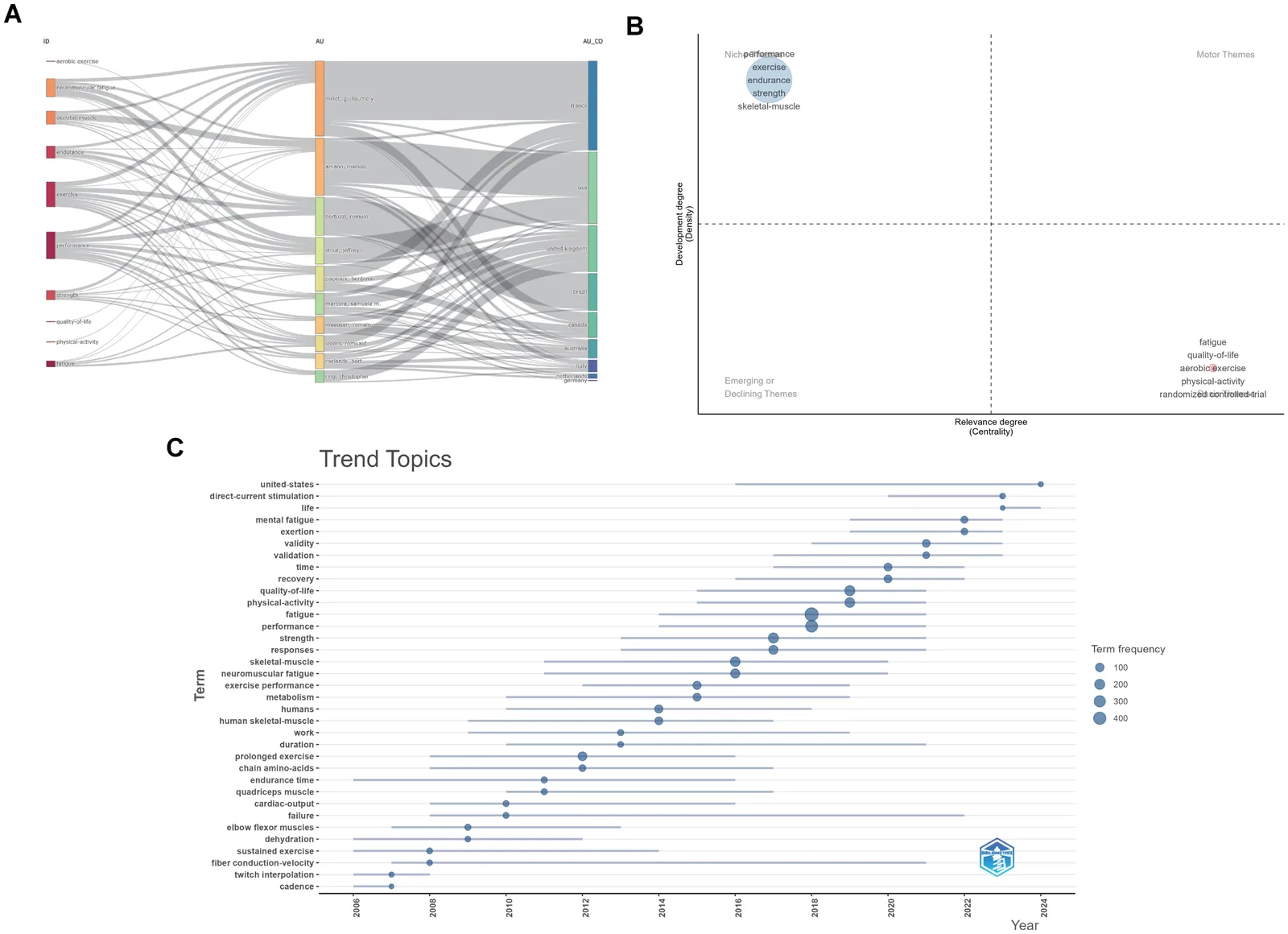
Analysis of topic evolution trends. (A) Sankey diagram. This figure depicts the relationships among keywords, authors, and countries, providing insights into the distribution and collaboration patterns within the research field. (B) Topic centrality plot. This figure visually represents the relative influence of different research topics, revealing the most important themes in the field. (C) Topic trend plot. This figure highlights the increasing attention and influence of specific topics, particularly “performance” and “mental fatigue,” over time.

The Sankey diagram illustrates the intricate relationships between topic keywords, authors, and countries, unveiling the distribution and collaboration patterns within the field of exercise science (Fig. 6A). From left to right, the diagram first lists keywords such as “aerobic exercise,” “neuromuscular fatigue,” and “skeletal-muscle,” which are linked by lines of varying thickness to central authors, such as “Millet, Guillaume Y.,” “Amann, Markus,” and “Bertuzzi, Romulo.” The width of the lines intuitively reflects the depth and influence of each author’s research in these specific areas. For example, “Millet, Guillaume Y.” stands out within the French research network, making significant contributions to “neuromuscular fatigue” and connecting to a variety of other keywords, indicating his broad research interests and substantial impact in the field. As the lines extend toward the right, they ultimately converge with the countries listed on the right, including the United States, the United Kingdom, and Brazil. This shows the international nature of collaboration and communication in exercise science research. It is notable that although some authors, such as “Millet, Guillaume Y.,” are primarily associated with France, their research influence extends across borders, intersecting with researchers from countries like the United States and the United Kingdom, reflecting the global trend of scientific cooperation.

The topic centrality plot clearly displays the relative influence of different topics in the research landscape through coordinates and point positions (Fig. 6B). Each point represents a research topic, with its location indicating the topic’s development level and relevance. Topics in the upper-left corner of the plot, such as “performance,” “exercise,” “endurance,” “strength,” and “skeletal-muscle,” demonstrate high development and relevance, indicating these are mature and significant areas of research. On the other hand, topics in the lower-right corner, such as “fatigue,” “quality of life,” “aerobic exercise,” “physical activity,” and “randomized controlled trial,” although highly relevant, show relatively low development and represent emerging or evolving research fields (Fig. 6B). Through the topic centrality plot, we gain an intuitive understanding of the status and knowledge structure of different research topics, unveiling the key research hotspots and future development trends in the field.

We then used the R-Bibliometrix package to generate a topic trend chart, where each point represents a topic, with the size indicating its level of attention and influence. The chart reveals that terms such as “performance,” “exercise,” “endurance,” and “strength” have steadily increased over the years, indicating that these topics have consistently remained at the core of endurance and fatigue research, particularly from 2014 onward. Meanwhile, terms like “mental fatigue,” “quality of life,” and “fatigue” began to significantly rise from 2016, reflecting a shift in focus toward the psychological aspects of fatigue and its impact on quality of life, with mental fatigue gaining increasing attention each year. Additionally, terms like “sustained exercise,” “cardiac output,” and “failure” show some fluctuations, indicating periods of increased interest in these areas, but without establishing a sustained growth trend, possibly due to specific research interests surrounding particular issues (Fig. 6C). Overall, by analyzing these trends, we can clearly observe the development of various research directions, particularly the continued growth in the areas of mental fatigue and quality of life, while also revealing the diversity and dynamic changes within the research landscape.

## 4. Discussion

### 4.1. Current status of the topic based on bibliometric analysis

Our study employed bibliometric methods to analyze research in the field of endurance exercise and central fatigue over the past 20 years, revealing the diversity and complexity within this domain. The findings demonstrate a steady increase in the number of publications, rising from 37 articles in 2005 to 171 in 2024, signaling a growing body of research and heightened activity. While fluctuations in publication numbers occurred in certain years, the overall trend reflects the increasing prominence of this field within sports science and physiology.

In terms of the evolution of research themes, core topics such as “performance,” “exercise,” “endurance,” and “strength” have seen a gradual rise since 2014, indicating the expanding focus on the effects of endurance exercise on athletic performance and physiological adaptation. Additionally, as research has deepened, themes concerning mental fatigue and quality of life have gained increasing attention, particularly the terms “mental fatigue” and “quality-of-life,” which saw significant growth after 2016. This shift highlights the growing recognition of the biological, psychological, and social impacts of fatigue.

The double-map overlay analysis further illustrates the strengthening interdisciplinary integration within the field. Citing journals are primarily concentrated in molecular biology, immunology, and clinical sciences, reflecting a gradual shift towards interdisciplinary collaboration in the study of fatigue mechanisms, particularly in the areas of neuroscience and nutrition.

Keyword analysis also underscores the interdisciplinary trend, with core keywords like “fatigue,” “exercise,” and “performance” forming tightly interconnected networks. Burst analysis of keywords revealed a marked increase in terms such as “prolonged exercise” and “human skeletal muscle,” which underscore the importance of prolonged exercise on central fatigue and the role of skeletal muscles in fatigue mechanisms. Notably, the term “mental fatigue” has seen rapid growth since 2015, further emphasizing the increasing interest in the psychological aspects of fatigue.

Collaboration network analysis demonstrates the critical role of international cooperation in advancing research in this field. Research institutions from countries such as the United States, the United Kingdom, Canada, and China have been instrumental in driving in-depth studies. Prominent institutions like the French National Institute for Health and Medical Research (INSERM) and Harvard University have played a key role in fostering interdisciplinary research integration.

Through multiple correspondence analysis and co-citation network analysis, we have identified key research areas and the cross-disciplinary nature of the field. Topics such as the relationship between endurance performance and central fatigue, neuromuscular fatigue, and the impact of resistance training on quality of life intersect, reflecting the diversity and interconnection within the field of sports science. Future research should continue to explore the biopsychosocial model, deepen interdisciplinary collaboration, and provide more comprehensive theoretical support for sports medicine and clinical practice.

We acknowledge that annual publication counts are count-data, and that Poisson regression, negative binomial regression, or segmented trend/joinpoint analysis may provide a more statistically appropriate framework than cubic polynomial fitting and univariate logistic regression for modeling potential overdispersion and structural breaks in the publication trend. The polynomial and logistic curves reported above (Fig. 2B,C) were used for descriptive visualization of the overall growth trajectory and its statistical association with time rather than for formal count-data inference; this is noted as a limitation of the trend-modeling approach, and future bibliometric studies in this field may adopt count-data regression methods to further validate the trend.

### 4.2. Neurotransmitters associated with endurance exercise and central fatigue

Central fatigue during endurance exercise is regulated by a complex interaction of various neurotransmitters. These neurotransmitters influence exercise performance, fatigue perception, mood, and motivation by modulating the brain and nervous system. NE plays a crucial role in exercise, particularly during endurance training, as it enhances attention, alertness, and psychological motivation to combat fatigue.^[34]^ Moreover, NE’s role in regulating the cardiovascular system helps improve exercise performance.^[35]^ During endurance training, levels of NE typically rise, delaying the onset of central fatigue.^[34]^ However, overtraining can deplete NE levels, reducing its functionality in the central nervous system and exacerbating fatigue.^[23]^

DA, a key neurotransmitter involved in regulating exercise motivation, pleasure, and reward mechanisms, can enhance positive emotions associated with exercise, thus boosting motivation.^[36]^ Endurance exercise increases DA levels, particularly after high-intensity activities, which helps alleviate fatigue and delay the onset of central fatigue.^[37]^ However, prolonged high-intensity exercise or overtraining can deplete DA, reducing motivation and increasing fatigue.^[38]^

5-HT plays an essential role in regulating mood, sleep, appetite, and cognitive functions. During endurance exercise, 5-HT synthesis and release are enhanced, which helps improve mood, boost performance, and alleviate fatigue. In endurance training, 5-HT levels tend to increase, particularly after prolonged exercise, improving the mood and fatigue tolerance of athletes. Low 5-HT levels are associated with mood disturbances and reduced motivation, which may exacerbate symptoms of central fatigue. Antagonizing 5-HT receptors in both humans and animal models has been shown to enhance endurance performance.^[39-41]^

Gamma-aminobutyric acid, as the primary inhibitory neurotransmitter, helps stabilize mood by inhibiting neural activation. Studies have shown that dietary supplementation with gamma-aminobutyric acid can enhance adaptive changes and endurance performance induced by training, possibly serving as an effective dietary supplement for athletes and individuals engaged in regular exercise.^[42]^ Glutamate, an excitatory neurotransmitter, is involved in memory, learning, and attention, enhancing the brain’s control over exercise. In endurance exercise, glutamate helps improve performance, but excessive release due to overtraining can cause neurotoxic reactions, exacerbating central fatigue.^[43]^

Acetylcholine is essential for motor control and muscle coordination, but central fatigue during endurance exercise may be linked to its depletion and decreased neurotransmission efficiency, negatively impacting performance.^[44]^ Endorphins, as natural analgesics, improve mood, alleviate pain, and reduce physiological discomfort during prolonged exercise, helping to mitigate central fatigue.^[45]^ Cortisol, which regulates energy balance and stress, can be beneficial during moderate exercise, but prolonged high-intensity exercise may elevate cortisol levels, further increasing fatigue.^[46]^ Oxytocin and brain natriuretic peptide play important roles in regulating fluid homeostasis, particularly under extreme physiological conditions following long-duration endurance exercise.^[47,48]^ Adenosine, an inhibitory neurotransmitter, regulates energy balance, sleep, and neural excitability. Its levels rise during endurance exercise, helping to attenuate neural excitability and delay fatigue.^[49]^

Endurance exercise impacts the balance of various neurotransmitters, which plays a significant role in the onset and progression of central fatigue.^[23]^ The equilibrium of neurotransmitters is crucial not only from a neurobiological perspective but also directly affects an athlete’s psychological state, motivation, and performance.^[50]^ Properly managing exercise intensity, training cycles, and incorporating appropriate recovery strategies are key to maintaining neurotransmitter balance and preventing central fatigue. Future research should explore the specific mechanisms of these neurotransmitters to provide new theoretical foundations and practical guidance for sports medicine and fatigue management.

### 4.3. Clinical implications

Endurance exercise plays a pivotal role in modulating the onset and progression of central fatigue through a combination of biological, psychological, and sociological mechanisms.^[50]^ As highlighted earlier, at the biological level, endurance exercise regulates neurotransmitters such as NE, DA, and 5-HT, which improve exercise performance and delay the perception of fatigue. DA enhances motivation and positive emotional states during exercise, while 5-HT helps mitigate negative emotions, regulate mood, and cope with stress, thereby alleviating central fatigue. Psychological states and social support systems also significantly contribute to the regulation of fatigue, as strengthening self-efficacy and social networks can ease fatigue perception.

The application of well-structured exercise periodization, balancing training intensity, duration, and recovery periods, is critical in preventing neurotransmitter imbalance induced by overtraining and promoting recovery. Recovery strategies such as cold-water immersion, massage therapy, and sleep interventions facilitate recovery by modulating neurotransmitter levels and enhancing resilience.^[51,52]^ Moreover, endurance exercise improves cognitive function, particularly in the context of high-intensity training or fatigue states, helping mitigate the negative impact of mental fatigue on cognitive performance.

In clinical practice, literature captured within this bibliometric mapping has proposed endurance exercise as a candidate non-pharmacological intervention for conditions such as chronic fatigue syndrome, depression, and anxiety. These proposed benefits derive from the cited mechanistic and clinical studies rather than from the present bibliometric analysis itself and warrant confirmation through independent mechanistic or clinical evidence before being regarded as established. It can enhance mood, reduce fatigue, and improve quality of life. Endurance exercise has also been shown to be effective in alleviating central fatigue in elderly populations, cancer patients, and individuals with cardiovascular disease. Future research should focus on further elucidating the impact of endurance exercise on neurobiological mechanisms, particularly neurotransmitter regulation and neuroprotective effects, and explore its therapeutic potential in addressing cognitive impairments and mental health disorders. Personalized exercise interventions and optimized training methods will provide stronger theoretical support for sports medicine and clinical practice, enhancing both the physical and psychological well-being of patients.

## 5. Conclusion

This study systematically analyzes the research trends, hotspots, and future directions in the field of endurance exercise and central fatigue using bibliometric methods. The findings from the past 2 decades indicate that neurotransmitter regulation is a recurring research theme associated with endurance exercise and central fatigue in the literature mapped by this analysis, alongside complex biological, psychological, and sociological dimensions of fatigue. As a bibliometric study, these associations should not be interpreted as direct evidence that endurance exercise causally modulates central fatigue, which requires confirmation by mechanistic and clinical research. Despite the continued growth in research quantity, the occurrence and exacerbation of central fatigue remain pressing challenges as exercise intensity increases. Future research should delve deeper into the neurobiological mechanisms of endurance exercise, particularly its effects on neurotransmitter regulation and neuroprotection, aiming to uncover its potential applications in clinical conditions such as chronic fatigue syndrome, mental health disorders, and cognitive impairments. Further individualized exercise interventions and evidence-based recovery strategies will offer a more robust theoretical foundation for sports medicine and promote the widespread application of endurance exercise in enhancing both physical and mental health.

## Acknowledgments

The authors thank VOS viewer, Cite Space the developers of VOSviewer and CiteSpace for their support. This study was supported by the National Natural Science Foundation of China (Grant No. 8240155735). The authors declare no competing interests.

## Author contributions

**Conceptualization:** Tie Li.

**Data curation:** Zhongming Li, Hanyuan Zhang, Chaojun Wang, Tie Li.

**Formal analysis:** Kaiqiang Zhang, Junyi Liu.

**Funding acquisition:** Tie Li.

**Software:** Kaiqiang Zhang, Jiazhen Cao.

**Supervision:** Tie Li.

**Visualization:** Kaiqiang Zhang.

**Writing – original draft:** Kaiqiang Zhang, Junyi Liu.

**Writing – review & editing:** Junyi Liu, Zhongming Li, Hanyuan Zhang, Chaojun Wang, Jiazhen Cao, Tie Li.
